# Assessing the impact of institution-specific guidelines for antimicrobials on doctors’ prescribing behavior at a German tertiary-care center and the additional benefits of providing a mobile application

**DOI:** 10.1371/journal.pone.0241642

**Published:** 2020-11-03

**Authors:** Sebastian G. Schönherr, Sebastian Wendt, Donald Ranft, Bettina Schock, Christoph Lübbert

**Affiliations:** 1 Division of Infectious Diseases and Tropical Medicine, Department of Medicine II, Leipzig University Hospital, Leipzig, Germany; 2 Institute for Medical Microbiology and Epidemiology of Infectious Diseases, Leipzig University Hospital, Leipzig, Germany; 3 Interdisciplinary Center for Infectious Diseases, Leipzig University Hospital, Leipzig, Germany; 4 Hospital Pharmacy, Leipzig University Hospital, Leipzig, Germany; 5 Institute for Hygiene, Hospital Epidemiology and Environmental Medicine, Leipzig University Hospital, Leipzig, Germany; 6 Department of Infectious Diseases/Tropical Medicine, Nephrology and Rheumatology, Hospital St. Georg, Leipzig, Germany; University of Sydney, AUSTRALIA

## Abstract

**Objective:**

To assess usage patterns, perceived usability, and effects of institution-specific guidelines (ISGs) for antimicrobials on clinicians’ prescribing behavior and the additional benefits of the mobile application (app), a single-center survey among medical doctors was performed.

**Methods:**

The study was carried out in a 1451-bed tertiary-care academic medical center in Leipzig, Germany. To ensure optimal empirical antibiotic therapies, appropriate diagnostics, and targeted antimicrobial prophylaxis, ISGs were provided as printed pocket guides (since 2014), a PDF version on ward computers, and a mobile app (since 2017). For the survey, we used an electronically structured cross-sectional questionnaire with 31 items, ordinal Likert scales, and percent bars, allowing for quantitative comparisons.

**Results:**

Of the 914 doctors contacted by email, 282 (31%) responded, and 254 (28%) surveys were eligible. ISGs were reported to be the most commonly used source of information for antimicrobial prescribing among the respondents. Ninety-four percent used ISGs at least once and 55% at least weekly. On average, participants reported using them in 38% of antibiotic prescriptions and to adhere to consulted recommendations in 87% of cases. Young clinicians (≤ 30 years) reported significantly higher use of the ISGs than their older colleagues (47% vs. 35% of antibiotic prescriptions, p = 0.004). Ninety-six percent of users found ISGs to be user-friendly, and nearly 100% recommended ISGs to other colleagues. Forty-five percent regarded the app as the most user-friendly way to access ISGs, and app users were significantly more likely to use ISGs regularly (p = 0.024). Eighty-four percent reported behavioral changes regarding at least one aspect of antimicrobial therapy (e.g. duration, application mode, prescription frequency), while 54% reported changes regarding the choice of specific substance groups.

**Conclusions:**

ISGs are used regularly and appear to have a relevant impact on clinicians’ prescribing habits. A mobile app may be the most effective way to provide ISGs, although multiple platforms seem to add value. While the majority of participants reported perceived effects on their prescribing behavior, this study does not allow any conclusions to be drawn about the extent of the effects of ISGs on antibiotic use and patient outcomes.

## Introduction

Institution-specific guidelines (ISGs) for antimicrobial agents are considered an important part of antibiotic stewardship (ABS) programs [1,2]. ISGs offer locally tailored decision support for empiric antimicrobial prescribing that reflects local pathogen and resistance patterns, thereby aiming to improve the quality of prescriptions, minimize unnecessary use of antimicrobials, lower side effects and optimize treatment outcomes [2]. The Leipzig University Hospital, Germany, first introduced ISGs in 2014 as a printed pocket guide and a PDF version for desktop computers. An electronic application (app) for smartphones and other mobile devices (based on the PDF version) was added two and a half years later.

To assess patterns of use, perceived usability, and effects of ISGs on clinicians’ prescribing behavior, as well as the additional benefits of the app, a survey was carried out among medical doctors to document the following information: (i) clinicians’ attitudes and behavior toward recommendations of ISGs, (ii) the impact of ISGs on the usage of antimicrobials by clinicians, (iii) the potential added value of the app version of the ISGs, and (iv) differences in ISG use between subgroups defined by medical specialization, age, sex, and exposure to regular ward rounds by an ABS team—which is composed of a clinical microbiologist, an infectious disease expert, and a clinical pharmacist. Regular ABS rounds are carried out in the following medical areas: neonatology, pediatric intensive care unit (ICU), surgical isolation ward, transplant surgery, gastroenterology/hepatology, pneumology, surgical ICU, medical ICU, and neurological ICU.

## Methods

### Study setting and design

The Leipzig University Hospital is a 1451-bed tertiary-care facility in Leipzig, Germany. The ISGs comprise 45 individual chapters, which cover infections from a wide range of clinical fields, and provide detailed information on empirical treatment, appropriate diagnostics, targeted antimicrobial prophylaxis as well as dose adjustments for patients with organ dysfunction. In addition, the most important hygiene standards of the hospital are listed. Moreover, the ISGs contain the contact details of local experts in infectious diseases/tropical medicine, clinical microbiology, clinical virology, clinical pharmacy, laboratory medicine, and hospital hygiene. The ISGs take into account current local pathogen and resistance patterns, as well as national guidelines and other higher-level recommendations. In addition to printed pocket guides (144 pages) and a PDF version for computers in the wards, an app for smartphones and other mobile devices was launched in January 2017, accompanied by intensive internal communication and public relations work. A search function as well as an interactive table of contents enable quick access to queried ISG content. Since its introduction, the app has been downloaded approximately 7,200 times by external users and more than 1,000 times by employees of the hospital. The app can be purchased through common app stores for 3.49 Euro and is available free of charge to hospital employees using promotional offer codes. The app was developed and maintained with the help of the start-up software company AppsolutEinfach® (Halle/Saale, Germany, https://www.appsoluteinfach.de/). The download price covers all expenses. Updates are conducted biannually for the pocket guide and the PDF version, and every 6 months for the app version. Further information can be found on a dedicated landing page: https://www.uniklinikum-leipzig.de/Seiten/app-antiinfektiva.aspx [3].

The study tool was a self-administered online survey, and the sample frame included all medical doctors in the hospital, as all clinicians can prescribe antibiotics. The survey questions were developed and reviewed by experts specialized in infectious diseases, hospital hygiene, and hospital pharmacy—including a psychologist and scientists from the Robert Koch Institute, Berlin, Germany. The survey was pretested among ten physicians from the department of pneumology. The online questionnaire, comprising a total of 31 items, was generated using SoSci Survey [4] and was made available to participants by providing a shared link to www.soscisurvey.de by e-mail (**S1 Table**). The study was conducted in May 2019, approximately five years after the release of the ISGs and two years after the introduction of the app. The initial target response rate was set at 50% but was lowered to 30% given the stagnation of responses. Based on our first review of the respondents’ data, reminders were sent out to departments with little participation in order to receive responses from at least 30% of physicians from medical specializations in which antimicrobial agents are regularly prescribed.

### Statistical analysis

Only completed surveys were included in the final analysis, although completion was reached with fewer answered questions for participants who had not yet used the ISGs or certain ISG platforms. We analyzed the data as a whole and stratified for different subgroups—including (a) sex, (b) age groups, (c) medical specializations, (d) app users, and (e) wards with routine rounds by the ABS team. Ordinal Likert scales and percent bars allowed for quantitative comparisons. The Chi-square test was used to analyze subgroup differences for ordinal scale questions, t-test and ANOVA were used to test for mean differences in the percent scale questions, and p-values < 0.05 were considered significant. All calculations were carried out using SPSS^®^ version 24.0 (IBM, Armonk, NY, USA).

### Ethics

The study was reviewed and approved on January 22, 2019, by the ethics committee of the Medical Faculty of Leipzig University (registry number 011/19-ek). Approval was obtained before the study began, particularly before the surveys were dispatched and data collected. All data were anonymous and could not be traced to the respondents. Before the survey, participants were given an explanatory text explaining the relevance of the project and the scientific use of the survey. Therefore, consent was assumed when the electronic questionnaire started.

## Results

### Participants and frequency of ISG use

**Fig 1:**
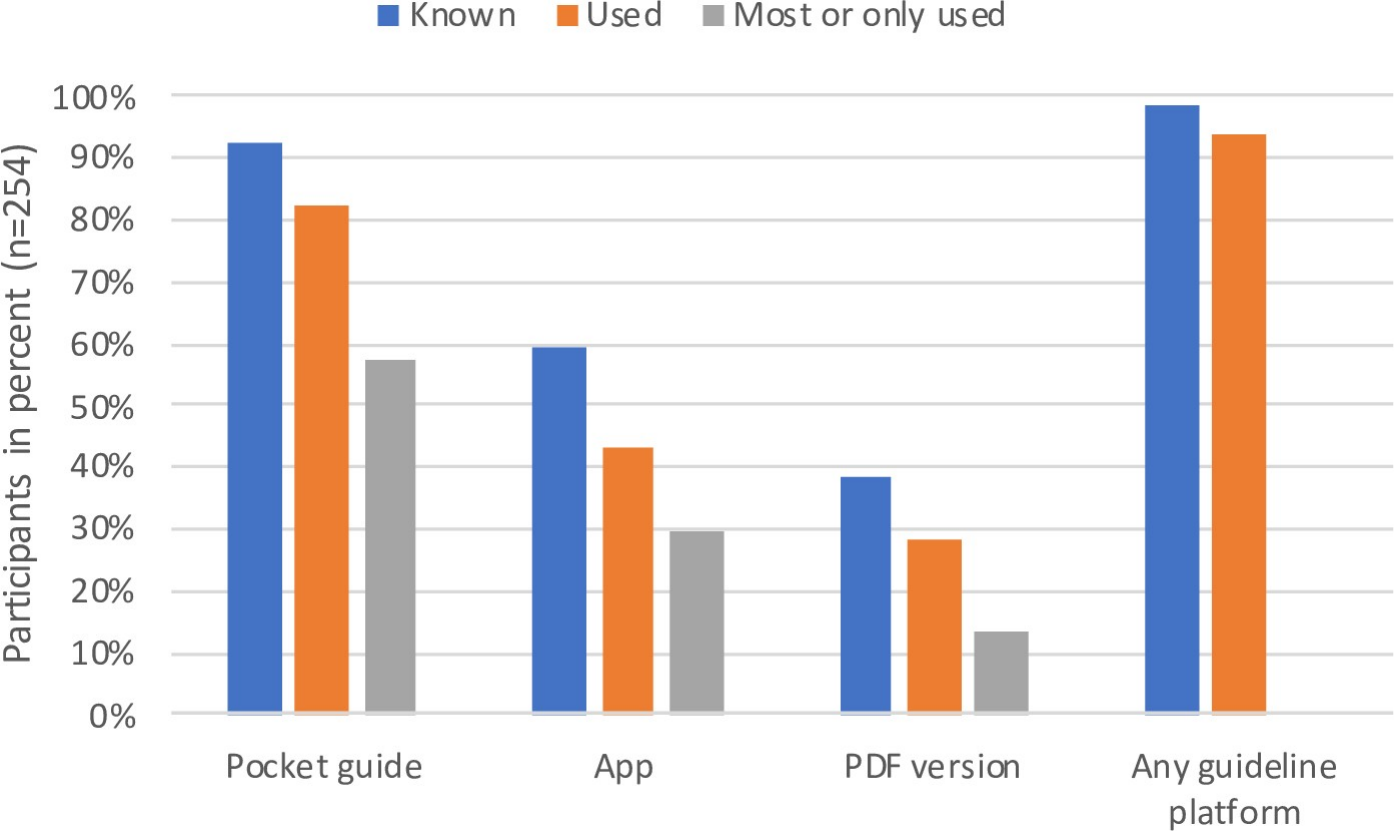
Knowledge and use of the different platforms of institution-specific guidelines. Data collected from survey items 1 and 2 (S1 Table).

Of 914 medical doctors contacted by e-mail, 282 (31%) responses were received, and 254 (28%) surveys were eligible for the analysis. Of the 28 excluded surveys, 26 were incomplete and 2 contained conflicting statements regarding a single item (question 2) of the survey. Therefore, the latter two were excluded from the analysis of this item but were included in all other analyses (**S1 Table**). Almost all participants (253/254) considered a strict indication for antibiotic use to be important (96% “very important” and 3.5% “rather important”). Nearly 100% of the participants (252/254) knew the ISGs, although only 150 (59%) were aware of the availability of the app format (**Fig 1**).

Ninety-four percent (238/254) of the respondents stated to have used ISGs at least once (referred to as users) and 55% (140/254) at least weekly (referred to as regular users; **Table 1**). On average participants reportedly consulted the ISGs in 38% of cases in which they prescribed antibiotics. The mean reported adherence rate to consulted ISGs was 87%. We expected to find differences in the patterns of ISG use between medical specializations and other subgroups and hypothesized that younger clinicians and those exposed to ward rounds by the ABS team would demonstrate more extensive use and better adherence to ISGs. When comparing age groups, we found that younger doctors (≤ 30 years) used ISGs for a higher proportion of their antibiotic prescriptions (47% vs. 35%, p = 0.004) and were more likely to use ISGs regularly than their older colleagues (73% vs. 50%, p = 0.002). Clinicians working in hospital areas with regular ward rounds by the ABS team were more likely to use the ISGs at least once per week than others (68% vs. 51%, p = 0.013), although they reported a lower adherence to the recommendations (90% vs. 81%, p = 0.003). Moreover, we found a range of sex differences: Males answered the survey more often than females (148 vs. 105) and used the app significantly more often (52% vs. 31%; p = 0.001). Female doctors, on the other hand, stated they used the ISGs for a larger proportion of their prescriptions (43% vs. 34%, p < 0.05) and reported higher adherence to ISGs (91% vs. 85%, p < 0.01) than males (**Table 1**). When considering age as a potential confounder for analyzing the frequency of ISG use in other subgroups, statistical effects remained significant even when younger participants (≤ 30 years) were excluded.

**Table 1:** Comparison of subgroups defined by age, sex, specialization, exposure to ABS ward rounds, and their preferred way of accessing the ISGs.

		Percent of regular ISG users (≥1x/week)			ISG use in percent of total antibiotic prescriptions			Adherence to consulted ISG recommendations		
Participant characteristics		All survey participants	Regular ISG users	P value	ISG users	Mean percentage	P value	ISG users	Mean percentage	P value
**Total**		254	140 (55%)		238	38%		238	87%	
**Age**	≤ 30	56	41 (73%)	**0.002**	53	47%	**p = 0.004**	53	88%	0.923
	> 30	198	99 (50%)		185	35%		185	87%	
**Sex**	Male	148	83 (56%)	0.777	138	34%	**p = 0.015**	138	85%	**0.001**
	Female	105	57 (54%)		99	43%		99	91%	
	Diverse*	1	0 (0%)	-	1	62%	-	1	84%	-
**ABS ward rounds**	Yes	66	45 (68%)	**0.013**	65	37%	p = 0.839	65	81%	**0.003**
	No	188	95 (51%)		173	38%		173	90%	
**Specialization**	Internal medicine	72	51 (71%)	**0.0003**	71	39%	p = 0.257	71	89%	0.546
	Anesthesia	41	28 (68%)		38	36%		38	84%	
	Surgery	41	18 (44%)		38	31%		38	86%	
	Other clinicians	89	37 (42%)		80	41%		80	87%	
	Non-clinical doctors*	11	6 (55%)	-	11	38%	-	11	94%	-
**ISG-platform most frequently used**	Pocket guide	133	67 (50%)	**0.024**	133	39%	p = 0.264	133	88%	0.955
	App	69	46 (67%)		69	39%		69	87%	
	PDF	31	22 (71%)		31	31%		31	87%	
	Excluded	5	-	-	5	-	-	5	-	-
**Number of used ISG platforms**	≥2	129	87 (67%)	**0.003**	129	36%	p = 0.203	129	90%	**0.014**
	1	109	53 (49%)		109	40%		109	84%	

The data was collected from survey items 12, 15 and 16 (S1 Table). An asterisk (*) symbolizes that the subgroup size was too small for statistical testing.

Abbreviations: ABS = antibiotic stewardship, ISGs = institution-specific guidelines, PDF = portable document format.

### ISG use and acceptance of content

We were curious to see which content of the ISGs was most relevant for the users and whether they agreed with the recommendations. The types of information looked up most frequently were those regarding the choice of substances (216/238, 91%), dosage (198/238, 83%), and therapy duration (137/238, 58%). No strong trends were noted regarding the types of cases that doctors would likely consult the ISGs: 49% (117/238) stated they used them in routine cases, 62% (148/238) for rare infections, 53% (125/238) for cases outside their specialization, and 45% (107/238) for pharmaceutical questions (e.g. dose adjustments; **Table 2**).

**Table 2:** Comparison of age groups regarding ISG use and acceptance of content.

Survey items and answers		Total	≤ 30 years	> 30 years	P value
	Number of survey respondents	238	53	185	
**Content of guideline used**					
	Choice of substance	216 (91%)	50 (94%)	166 (90%)	0.307
	Antibiotic dose	198 (83%)	50 (94%)	148 (80%)	**0.014**
	Duration of therapy	137 (58%)	41 (77%)	96 (52%)	**0.001**
	Diagnostic procedures	13 (5%)	5 (9%)	8 (4%)	-
	Hygiene measures	21 (9%)	4 (8%)	17 (9%)	-
	Antimicrobial prophylaxis	34 (14%)	10 (19%)	24 (13%)	0.280
	Other information	10 (4%)	1 (2%)	9 (5%)	-
	≥3 stated	140 (59%)	42 (79%)	98 (53%)	**0.001**
**Situations of guideline use**					
	Routine cases	117 (49%)	39 (74%)	78 (42%)	**0.0001**
	Rare infections	148 (62%)	33 (62%)	115 (62%)	0.989
	Infections outside the specialization	125 (53%)	30 (57%)	95 (51%)	0.500
	Pharmaceutical questions	107 (45%)	29 (55%)	78 (42%)	0.105
	Other situations	12 (5%)	3 (6%)	9 (5%)	-
	≥3 stated	79 (33%)	28 (53%)	51 (28%)	**0.0004**

Abbreviations: ISGs = institution-specific guidelines.

When comparing subgroups, we found that younger doctors were more likely to use different types of information provided by the ISGs (mean 3.04 vs. 2.53 items; p = 0.001) and that they used ISGs in a broader range of situations (mean 2.53 vs. 2.03 items, p < 0.001) than their older colleagues (**Table 2**). In particular, younger doctors were more likely to use ISGs’ information on dosage and therapy duration. Overall, agreement with the content of the guidelines was high: 89% (212/238) of the users agreed with all of the recommendations of the ISGs. There was disagreement on a few points of content, most frequently regarding the treatment of intracranial infections (e.g. the need for more specific content of it, **S1 and S3 Tables**). A frequently raised point to improve the content of ISGs was that recommendations regarding oral treatment alternatives should be extended. Other participants mentioned the need for more content on dose adjustment in specific patient groups (e.g. patients with continuous renal replacement therapy and obese patients; **S1 and S2 Tables**).

### Perceived usability and comparison of different information platforms

**Fig 2:**
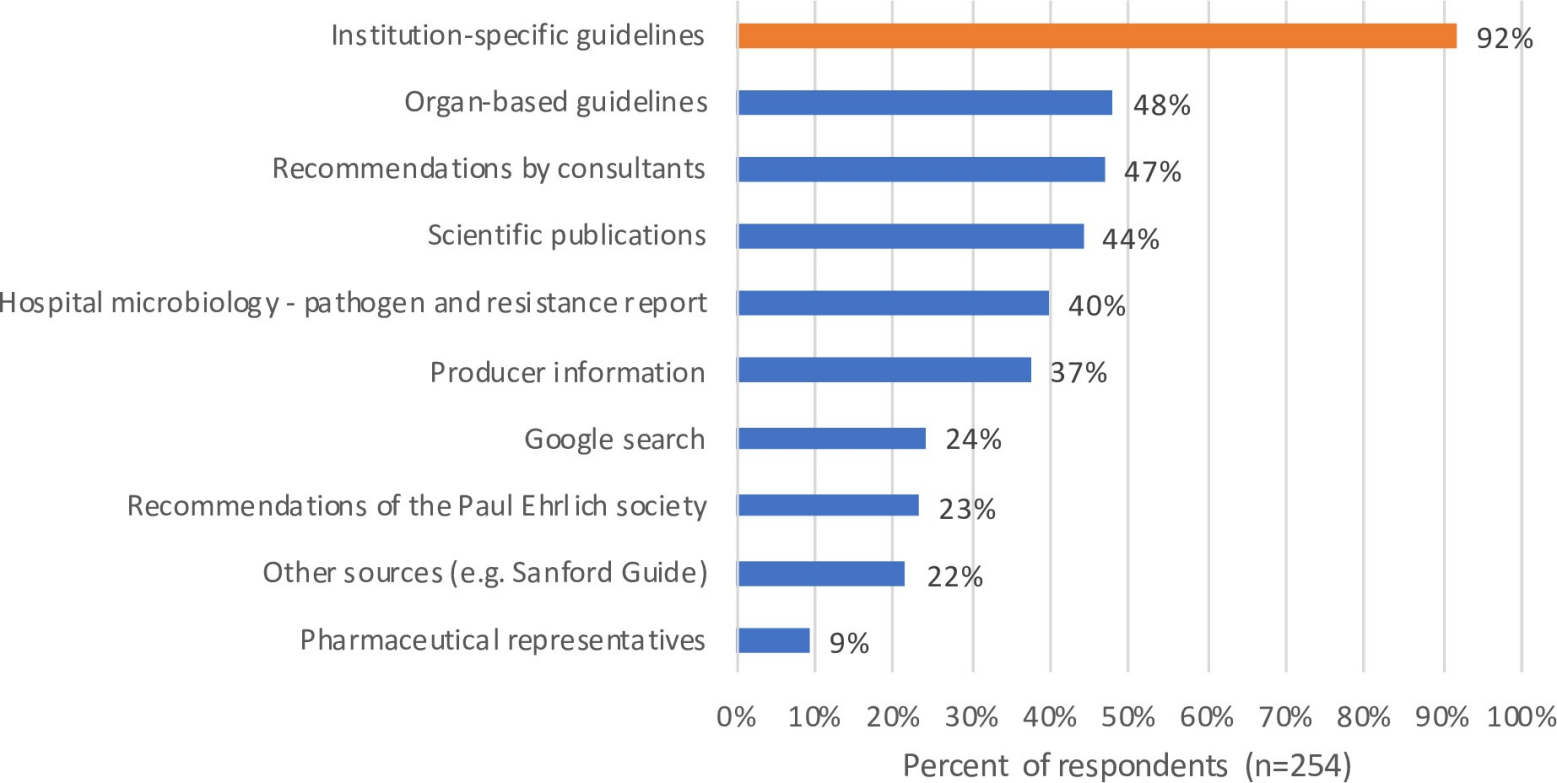
Use of ISGs in comparison with other information sources on empirical antimicrobial prescribing. Data collected from survey item 9 (S1 Table).

When compared to other sources of information for the empirical treatment of infections, ISGs were reported to be the most widely used tool among the participants: 233 participants (92%) reported using ISGs to find up-to-date information (**Fig 2**).

**Fig 3:**
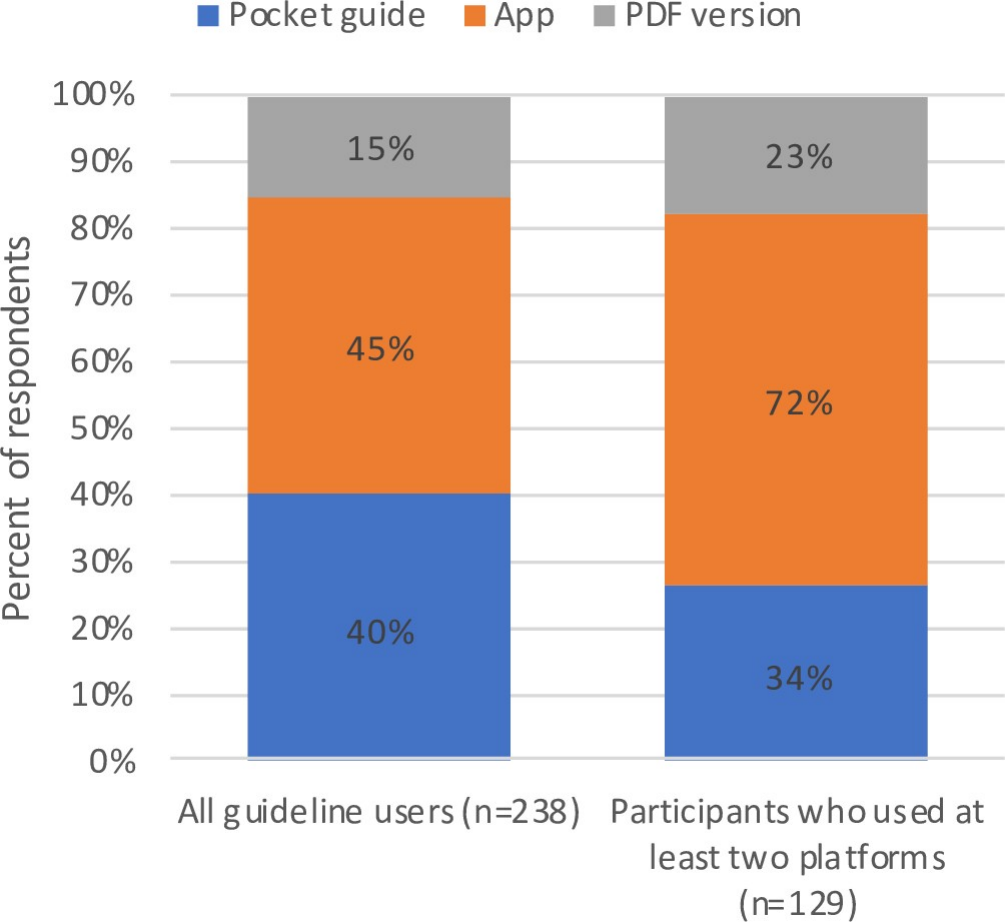
Responses regarding the most user-friendly way to access ISGs. Participants who had used at least two of the three available information platforms were significantly more likely to regard the mobile app as the most user-friendly option (p < 0.001). Data collected from survey items 2 and 26 (S1 Table).

Among 238 users, 228 (96%) found ISGs to be user-friendly, while 237 (99.6%) reported they would recommend ISGs to other colleagues. Ninety-three percent of the app users (102/110) stated the app was functioning well (52%) or rather well (41%) on their mobile devices. When asked which platform would be preferred in daily routine work, the app was the most preferred among the respondents (app: 45%, pocket guide: 40%, PDF version: 15%). This effect was stronger among the 129 participants who had already used ISGs on more than one platform (app: 72%, pocket guide: 34%, PDF version: 23%; **Fig 3**), and these participants were also more likely to use ISGs more than once a week than those who used only a single platform (67% vs. 49%, p = 0.003).

When comparing ISG users by platform use, we found significant differences between the three groups: Regular use of ISGs was more common among participants using predominantly the app (46/69; 67%) or the PDF version (22/31; 71%) than among those using the printed pocket guide (67/133; 50%) (p = 0.024). Furthermore, app users were more likely to consult ISGs for pharmaceutical questions (e.g. dose adjustments; p = 0.013) and decisions on therapy duration (p = 0.014) than others.

### Perceived effects on clinicians’ prescribing behavior

**Fig 4:**
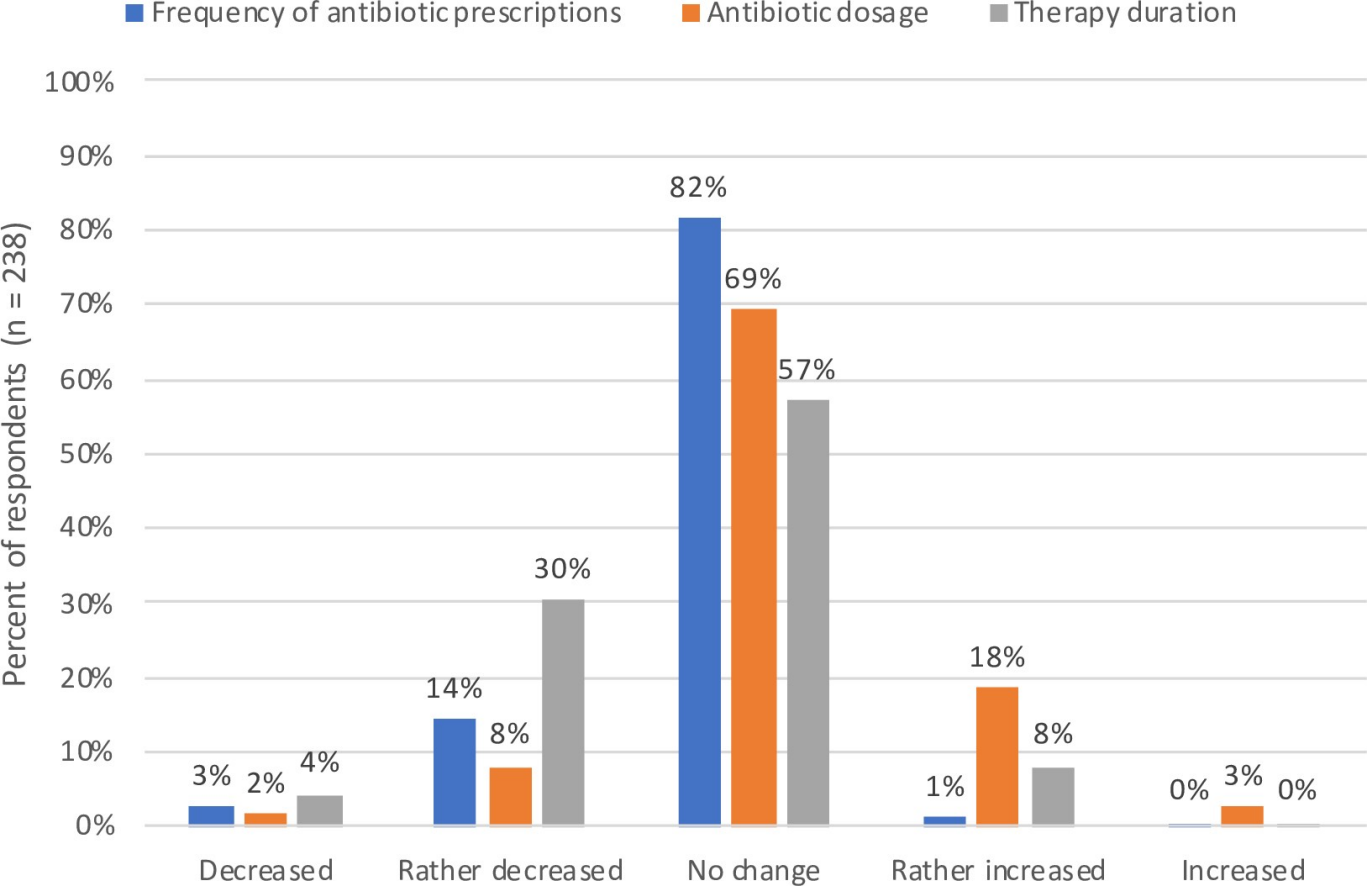
Perceived changes in prescribing behavior regarding dosage, therapy duration, and overall frequency of prescriptions due to the use of ISGs. Data collected from survey items 21 to 23 (S1 Table).

As ISGs are expected to lead to better therapeutic decisions by clinicians, we hypothesized that ISG users would be able to observe such changes in their behavior. Eighty-four percent (201/238) of the users reported at least one perceived effect on their prescribing habits. Fifty-four percent (128/238) stated they changed their prescribing behavior with regard to at least one specific group of substances. Sixty-two percent (148/238) indicated changes in their prescribing habits regarding the dosage of antimicrobial agents, therapy duration, application mode, or prescription frequency. For each of these items the majority stated no perceived change, although the changes that were stated show a tendency toward a shorter duration of antibiotic therapy (82/238, 34%), higher dosage (50/238, 21%), and less frequent prescriptions of certain antibiotics (40/238, 17%), particularly fluoroquinolones (**Fig 4 and S1 Table**).

Sixteen percent of the users (37/238) stated they were more likely to prescribe sequential (intravenous followed by oral) antibiotic therapies. Indications for antimicrobial therapy, for which changes in the respondents’ prescribing behavior were reported most frequently were respiratory infections (56/238, 24%), perioperative prophylaxis (18%), sepsis (43/238, 18%) and therapy of asymptomatic bacteriuria (37/238, 16%) (**Table 3**). Only one third (79) of the participants stated that their prescribing behavior had not changed in any situation.

**Table 3:** Summary of reported effects of ISGs on the prescribing behavior.

Perceived effects of the ISGs on prescribing behavior (n = 238)		No. (%)
**Antimicrobial substance groups for which the prescribing behavior changed due to the use of ISGs**		
	Fluoroquinolones	74 (31%)
	Second and third generation cephalosporins	67 (28%)
	Broad-spectrum penicillins	43 (18%)
	Carbapenems	43 (18%)
	Other antibiotics	25 (11%)
	Antifungals	15 (6%)
	Antivirals	8 (3%)
	At least one substance group stated	128 (54%)
**Situations in which behavior changed due to the use of ISGs**		
	Perioperative prophylaxis	44 (18%)
	Wound management	22 (9%)
	Sepsis therapy	43 (18%)
	Therapy of asymptomatic bacteriuria	37 (16%)
	Antibiotic therapy of respiratory infections	56 (24%)
	Blood culture collection	16 (7%)
	Other diagnostic procedures	14 (6%)
	Others	35 (15%)
	At least one situation stated	159 (67%)
**Aspects of antibiotic prescribing affected by the ISGs**		
	Frequency	44 (18%)
	Dosage	73 (31%)
	Therapy duration	102 (42%)
	Mode of application	37 (15%)
	De- or increase for at least one aspect stated.	148 (62%)
**Any change of the above items**	**201 (84%)**

Abbreviations: ISGs = institution-specific guidelines.

## Discussion

ISGs belong to the core elements of ABS programs and are considered to be a cornerstone in ensuring optimal outcomes of empirical antimicrobial therapy [2,5–7]. Smartphone apps have become increasingly popular in recent years and can serve as a platform for locally tailored support for clinical decision-making [8–16]. We found that the ISGs designated for the Leipzig University Hospital were successfully implemented, well liked, widely used, and well adhered to. We consider the mean reported usage rate of 38% of antibiotic prescriptions to be relatively high as guidelines for empirical treatment are not always applicable (e.g. when the pathogen is already specified or when complicating factors require a more individualized approach). We found significant differences in the frequency of ISG use between different medical specializations (i.e. higher frequency of use in internal medicine and anesthesia than in surgery and other fields) and in areas of the hospital with regular ward rounds by the ABS team, although we assume that these differences mainly reflect the different types of clinical cases that clinicians face in their respective specializations. Similarly, we expect that the lower adherence rates reported on wards with regular rounds of the ABS team are due to the higher prevalence of complicated infections in those areas (e.g. intensive care units) where the ISGs might be less applicable. ISGs appear to be particularly useful for young doctors with limited clinical experience, as they use them more extensively, although the fact that the ideal antibiotic therapy changes over time and updates are made frequently makes it relevant for everyone to consider locally tailored recommendations. In fact, other studies have suggested that antibiotic guidelines may be helpful for younger doctors to break away from the potentially harmful prescribing traditions of their superiors [15]. Therefore, we considered it positive that the ISGs of the Leipzig University Hospital have been valued across all age groups.

ISGs appeared to be the most important information source for the empirical treatment of infections in the Leipzig University Hospital, which is consistent with studies conducted in other institutions [17]. The provision of a mobile app appears to be of additional value: Although 41% of respondents were unaware of the availability of an app, it was still regarded as the most user-friendly way to access ISGs, yet the difference to the printed pocket guide was rather small. Furthermore, app and PDF users were more likely to consult the ISGs regularly. Potential reasons could be that digital versions make it easier to search for the information needed and, at least in the case of the app, are always available and up to date. This finding is consistent with a recent study in the UK that reported increased use of ISGs after moving from a pocket guide to a smartphone app [15]. Aside from being modern and trendy, digital platforms offer some practical advantages for the providers, as they can be easily distributed and updated regularly, with less risk that outdated recommendations will continue to be used. We therefore particularly recommend promoting app-based ISGs, although it is known that providing an app alone is not enough to achieve high levels of prescriber adherence [16].

There are many aspects that have to be considered for optimal antibiotic treatment, ranging from the correct indication to the right substance, mode of application, dosage and therapy duration. For each individual aspect, a majority of our study participants stated no perceived changes in their prescribing behavior, which is to be expected. When combining several aspects, however, we found that a large majority of ISG users had perceived an impact on their prescribing behavior in some respect. While the changes needed to improve the quality of antibiotic prescriptions vary by cases and prescribers, there have been some broader trends that were aimed for when implementing the ISGs such as higher doses, shorter durations, less use of fluoroquinolones and increased use of sequential therapy. In tendency, the perceived changes that were stated do meet these intentions, most prominently with regards to the duration of antibiotic therapy, with a third of ISG users reporting this impact.

The ISGs are particularly relevant for very common infections. In areas where overprescription is expected, participants reported perceived desired effects on their prescribing behavior (e.g. respiratory tract infections, urinary tract infections, and perioperative prophylaxis). Given the nature of our study tool, these results are only qualitative and rather subjective, although they provide valuable insight into the clinician’s perspective and show that the recommendations are being taken up, implemented and clinically worked on by them, thereby influencing various aspects of antimicrobial prescribing.

### Limitations

Our study has several limitations. First, regular ISG users might have been more willing to respond and could therefore be overrepresented. Second, participants might be biased toward socially desired answers, although—taking into account that the questionnaire was anonymous—we do not expect a strong effect here. Third, the study was conducted five years after the implementation of the ISGs. Therefore, in many cases, younger participants could not see behavioral changes due to the use of ISGs that they have used since the beginning of their careers. Thus, the frequency of use in combination with the adherence can provide more objective information. Fourth, only the perceived effects were recorded in the questionnaire. Hence, it cannot be said with certainty whether ISGs actually influenced the prescription of antibiotics. Fifth, there are several biases that make the use of the app more likely: The ISGs were available as pocket guides three years prior to the introduction of the app, and the app is reportedly less known than the pocket guide. All doctors automatically receive the printed pocket version when they start working. The app is not available for free in the app store, but it is said to be free for hospital employees by using promotional offer codes, although the comments in our survey show that many have not made use of this option. We believe that these factors might have reduced the preference for the app.

## Conclusions

The increased threat of antimicrobial resistance makes prudent and focused use of antibiotic substances an imperative priority. This study shows that ISGs may play an important role in optimizing antibiotic therapy as clinicians’ prescribing behavior appears to have been affected by them in a meaningful way. The ISGs of the Leipzig University Hospital are well-accepted and used across a wide range of medical specializations. They appear to be the most widely used resource for the empirical treatment of infections. ISGs seem to be particularly useful to young clinicians who use them most extensively. However, it is important that ISGs are used in all demographics and that they are valued and accepted by experienced clinicians as well. An app for mobile devices offers added value in the provision of policy information, as this might be preferred and used more frequently than the printed alternative due to several practical advantages for providers and users. The provision of multiple platforms may be beneficial as it offers additional improvements in acceptance and might lower the threshold of using the ISGs. While the majority of participants reported perceived effects on their prescribing behavior as a result of using the ISGs, this study does not allow any conclusions to be drawn about the extent of the effects of the ISGs on antibiotic use and patient outcomes.

## Supporting information

S1 Table Survey questions and answers. The original survey and answers in German can be obtained on request.
(DOCX)

S2 Table Written comments of survey participants regarding disagreement with ISG recommendations. Answers to question 17, translated from German.
(DOCX)

S3 Table Written comments of survey participants regarding the ISGs or the study. Answers to question 31 (free text, translated from German).
(DOCX)

S1 Additional figures (DOCX)
