# Supplementary material for: Assessing the impact of institution-specific guidelines for antimicrobials on doctors’ prescribing behavior at a German tertiary-care center and the additional benefits of providing a mobile application
Source: PLoS One. 2020 Nov 3;15(11):e0241642. doi: 10.1371/journal.pone.0241642 (PMC7608892; doi:10.1371/journal.pone.0241642)
Supplement: S1 Table — The original survey and answers in German can be obtained on request. (DOCX) [file pone.0241642.s001.docx]

**Supporting information**

| **S1 Table. Survey items and answers** | | | | | |
| --- | --- | --- | --- | --- | --- |
| **Questions and answers No. of respondents n (%)** | | | | | |
| **1. Do you know the institution-specific guidelines (ISGs) of Leipzig University Hospital?** | 254 | | |  |  |
| Yes |  | | | 252 | 99% |
| No |  | | | 2 | 1% |
| **If yes, which guideline platforms do you know?** |  | | |  |  |
| Pocket guide |  | | | 235 | 93% |
| App |  | | | 150 | 59% |
| PDF |  | | | 98 | 39% |
| **2. Have you ever used the ISGs of Leipzig University Hospital?** | 254 | | |  |  |
| Yes |  | | | 238 | 94% |
| No |  | | | 16 | 6% |
| **If yes, on which platform?** |  | | |  |  |
| Pocket guide |  | | | 208 | 82% |
| App |  | | | 110 | 43% |
| PDF on ward computers |  | | | 72 | 28% |
| **If several, which one most commonly?** | 195 | | |  |  |
| Pocket-guide most |  | | | 99 | 51% |
| App most |  | | | 65 | 33% |
| PDF most |  | | | 31 | 16% |
| **Summary: used most or alone** | 233 | | |  |  |
| Pocket-guide |  | | | 133 | 57% |
| App |  | | | 69 | 30% |
| PDF on ward computers |  | | | 31 | 13% |
| **3. Sex** | 254 | | |  |  |
| Male |  | | | 148 | 58% |
| Female |  | | | 105 | 41% |
| Diverse |  | | | 1 | 0,4% |
| **4. Age** | 254 | | |  |  |
| ≤ 25 |  | | | 1 | 0,4% |
| 26 - 30 |  | | | 55 | 22% |
| 31 - 40 |  | | | 126 | 50% |
| 41 - 50 |  | | | 55 | 22% |
| 51 - 60 |  | | | 13 | 5% |
| ≥ 61 |  | | | 4 | 2% |
| **5. How many years have you been working at Leipzig University Hospital?** | 254 | | |  |  |
| < 1 |  | | | 22 | 9% |
| 1 - 2 |  | | | 48 | 19% |
| 3 - 5 |  | | | 65 | 26% |
| 6 - 10 |  | | | 67 | 26% |
| 11 - 15 |  | | | 30 | 12% |
| 16 - 25 |  | | | 18 | 7% |
| 26 - 35 |  | | | 3 | 1% |
| ≥ 36 |  | | | 1 | 0,4% |
| **6. What is your specialization/which field are you specializing in?** | 254 | | |  |  |
| General medicine |  | | | 3 | 1% |
| Anesthesia |  | | | 41 | 16% |
| Occupational medicine |  | | | 4 | 2% |
| Ophthalmology |  | | | 2 | 1% |
| General surgery |  | | | 4 | 2% |
| Pediatric surgery |  | | | 6 | 2% |
| Orthopedics and trauma surgery |  | | | 14 | 6% |
| Plastic and aesthetic surgery |  | | | 2 | 1% |
| Visceral surgery |  | | | 8 | 3% |
| Obstetrics and gynecology |  | | | 6 | 2% |
| Ear, nose & throat |  | | | 6 | 2% |
| Skin and venereal diseases |  | | | 11 | 4% |
| Hygiene and environmental medicine |  | | | 2 | 1% |
| Internal medicine |  | | | 14 | 6% |
| Internal medicine, angiology |  | | | 6 | 2% |
| Internal medicine, endocrinology and diabetology |  | | | 7 | 3% |
| Internal medicine, gastroenterology |  | | | 12 | 5% |
| Internal medicine, hematology and oncology |  | | | 11 | 4% |
| Internal medicine, infectious diseases |  | | | 1 | 0,4% |
| Internal medicine, cardiology |  | | | 6 | 2% |
| Internal medicine, nephrology |  | | | 7 | 3% |
| Internal medicine, pneumology |  | | | 4 | 2% |
| Internal medicine, rheumatology |  | | | 4 | 2% |
| Pediatrics |  | | | 9 | 4% |
| Child and adolescent psychiatry |  | | | 3 | 1% |
| Microbiology, virology and epidemiology of ID |  | | | 7 | 3% |
| Oral and maxillofacial surgery |  | | | 3 | 1% |
| Neurosurgery |  | | | 4 | 2% |
| Neurology |  | | | 15 | 6% |
| Nuclear medicine |  | | | 3 | 1% |
| Psychiatry and psychotherapy |  | | | 7 | 3% |
| Psychosomatic medicine and psychotherapy |  | | | 1 | 0,4% |
| Radiology |  | | | 1 | 0,4% |
| Radiotherapy |  | | | 3 | 1% |
| Transfusion medicine |  | | | 1 | 0,4% |
| Urology |  | | | 5 | 2% |
| Dentistry |  | | | 7 | 3% |
| Pathology |  | | | 1 | 0,4% |
| Laboratory medicine |  | | | 1 | 0,4% |
| Other specializations |  | | | 1 | 0,4% |
| Emergency medicine |  | | | 1 | 0,4% |
| **Summary:** |  | | |  |  |
| Clinicians |  | | | 243 | 96% |
| Anesthesia |  | | | 41 | 16% |
| Surgery |  | | | 41 | 16% |
| Internal medicine |  | | | 72 | 28% |
| Other clinical specializations |  | | | 89 | 35% |
| Nonclinical specializations |  | | | 11 | 4% |
| **7. Are you working on a high-risk-ward as declared by the department of hospital hygiene (e.g. ICU, neonatology, transplant surgery, etc.)** | 254 | | |  |  |
| Yes |  | | | 86 | 34% |
| No |  | | | 168 | 66% |
| **8. Are you working on a ward with regular rounds by the antibiotic stewardship (ABS) team?** | 254 | | |  |  |
| Yes |  | | | 66 | 26% |
| No |  | | | 188 | 74% |
| **9. Where do you get up to date information regarding the use of antimicrobials?** | 254 | | |  |  |
| ISGs |  | | | 233 | 92% |
| Organ based guidelines |  | | | 122 | 48% |
| Recommendation by consultants |  | | | 119 | 47% |
| Scientific publications |  | | | 112 | 44% |
| Institution-specific pathogen and resistance report |  | | | 101 | 40% |
| Manufacturer’s information |  | | | 95 | 37% |
| Google search |  | | | 61 | 24% |
| Recommendations of the Paul Ehrlich Society |  | | | 59 | 23% |
| Other sources (e.g. Sanford Guide) |  | | | 55 | 22% |
| Pharmaceutical representatives |  | | | 24 | 9% |
| **10. How did you first hear about the ISGs?** | 252 | | |  |  |
| At the introduction orientation when starting my job |  | | | 66 | 26% |
| In a lecture |  | | | 6 | 2% |
| In my practical year |  | | | 17 | 7% |
| Recommendation of my colleagues |  | | | 47 | 19% |
| By chance |  | | | 3 | 1% |
| I was looking for such a tool |  | | | 2 | 1% |
| Through the antimicrobial commission of the hospital |  | | | 63 | 25% |
| On the hospital website |  | | | 24 | 10% |
| Other |  | | | 24 | 10% |
| **11. In your opinion, how important is a strict indication for antimicrobial therapy?** | 254 | | |  |  |
| Unimportant |  | | | 0 | 0% |
| Rather unimportant |  | | | 1 | 0,4% |
| Rather important |  | | | 9 | 4% |
| Important |  | | | 244 | 96% |
| No opinion |  | | | 0 | 0% |
| **12. How often do you use the ISGs?** | 238 | | |  |  |
| 1 × per year |  | | | 13 | 5% |
| 1 × per month |  | | | 85 | 36% |
| 1 × per week |  | | | 56 | 24% |
| several times per week |  | | | 69 | 29% |
| daily |  | | | 9 | 4% |
| several times per day |  | | | 6 | 3% |
| **13. Which types of information of the ISGs are you most likely to use?** | 238 | | |  |  |
| Choice of substance |  | | | 216 | 91% |
| Antibiotic dose |  | | | 198 | 83% |
| Duration of therapy |  | | | 137 | 58% |
| Diagnostic procedures |  | | | 13 | 5% |
| Hygiene measures |  | | | 21 | 9% |
| Antimicrobial prophylaxis |  | | | 34 | 14% |
| Other information |  | | | 10 | 4% |
| **14. In which situation are you most likely to use the ISGs? (several answers possible)** | 238 | | |  |  |
| Routine cases |  | | | 117 | 49% |
| Rare infections |  | | | 148 | 62% |
| Infections outside the specialization |  | | | 125 | 53% |
| Pharmaceutical questions |  | | | 107 | 45% |
| Other situations |  | | | 12 | 5% |
| **15. In what percentage of cases do you use the ISGs when prescribing antimicrobials?** | 238 | | |  |  |
| Summary of original data: |  | | |  |  |
| 1-10% |  | | | 50 | 21% |
| 11-20% |  | | | 37 | 16% |
| 21-30% |  | | | 33 | 14% |
| 31-40% |  | | | 24 | 10% |
| 41-50% |  | | | 29 | 12% |
| 51-60% |  | | | 12 | 5% |
| 61-70% |  | | | 18 | 8% |
| 71-80% |  | | | 17 | 7% |
| 81-90% |  | | | 8 | 3% |
| 91-100% |  | | | 10 | 4% |
| **16. In what percentage of cases are you adhering to the recommendations when you consulting the ISGs?** | 238 | | |  |  |
| Summary of original data: |  | | |  |  |
| 1-10% |  | | | 4 | 2% |
| 11-20% |  | | | 2 | 1% |
| 21-30% |  | | | 0 | 0% |
| 31-40% |  | | | 2 | 1% |
| 41-50% |  | | | 5 | 2% |
| 51-60% |  | | | 2 | 1% |
| 61-70% |  | | | 8 | 3% |
| 71-80% |  | | | 28 | 12% |
| 81-90% |  | | | 67 | 28% |
| 91-100% |  | | | 120 | 50% |
| **17. Are there any recommendations in the ISGs that you do not agree with?** | 238 | | |  |  |
| No |  | | | 212 | 89% |
| Yes |  | | | 26 | 11% |
| If yes, which are they? (For complete answers see S2 Table.) | | | | | |
| **Frequently mentioned recommendations/ topics:** | |  | |  |  |
| *Meningitis* | | |  | 5 |  |
| *Intracranial infections/ neurosurgical infections (other than meningitis)* | | |  | 3 |  |
| *Urinary tract infections (choice of substance/ oral alternatives)* | | |  | 3 |  |
| *Antibiotic coverage of enterococci in abdominal infections* | | |  | 2 |  |
| *Adnexitis recommendation* | | |  | 2 |  |
| *Spondylodiscitis recommendation* | | |  | 2 |  |
| *Dosage in zoster opthalmicus therapy* | | |  | 2 |  |
| **18. Would you recommend the ISGs to your colleagues?** | 238 | | |  |  |
| Yes |  | | | 237 | 99,6% |
| No |  | | | 1 | 0,4% |
| **19. Has your prescribing behavior changed due to the ISGs? If so, for which substance groups?** | 238 | | |  |  |
| Fluoroquinolones |  | | | 74 | 31% |
| Second & third generation cephalosporins |  | | | 67 | 28% |
| Broad-spectrum penicillins |  | | | 43 | 18% |
| Carbapenems |  | | | 43 | 18% |
| Other antibiotics |  | | | 25 | 11% |
| Antifungals |  | | | 15 | 6% |
| Antivirals |  | | | 8 | 3% |
| My prescribing behavior has not changed due to the guidelines |  | | | 110 | 46% |
| **20. In which of the following situations has your behavior changed due to the ISGs? (several answers possible)** | 238 | | |  |  |
| Perioperative prophylaxis |  | | | 44 | 18% |
| Wound management |  | | | 22 | 9% |
| Sepsis therapy |  | | | 43 | 18% |
| Therapy of asymptomatic bacteriuria |  | | | 37 | 16% |
| Antibiotic therapy of respiratory infections |  | | | 56 | 24% |
| Blood culture collection |  | | | 16 | 7% |
| Other diagnostic procedures |  | | | 14 | 6% |
| None |  | | | 79 | 33% |
| Other |  | | | 35 | 15% |
| **21. Due to ISGs recommendations the frequency of my antibiotic prescriptions has overall…** | 238 | | |  |  |
| Decreased |  | | | 6 | 3% |
| Rather decreased |  | | | 34 | 14% |
| Not changed |  | | | 194 | 82% |
| Rather increased |  | | | 3 | 1% |
| Increased |  | | | 1 | 0% |
| **Examples for increased prescriptions (optional):** | 34 | | |  |  |
| *Cephalosporins* |  | | | 20 |  |
| *Broad-spectrum penicillins* |  | | | 9 |  |
| *Piperacillin/Tazobactam* |  | | | 5 |  |
| *Carbapenems* |  | | | 3 |  |
| *Fosfomycin* |  | | | 3 |  |
| *Clarithromycin* |  | | | 2 |  |
| *Clindamycin* |  | | | 1 |  |
| **Examples for decrease of prescriptions (optional):** | 32 | | |  |  |
| *Fluoroquinolones* |  | | | 18 |  |
| *Broad-spectrum penicillins* |  | | | 4 |  |
| *Carbapenems* |  | | | 4 |  |
| *Cephalosporins* |  | | | 4 |  |
| *Piperacillin/Tazobactam* |  | | | 1 |  |
| *Linezolid* |  | | | 1 |  |
| *Cotrimoxazol* |  | | | 1 |  |
| **22. Due to the ISGs recommendations the antimicrobial doses you are prescribing have in tendency…** | 238 | | |  |  |
| Decreased |  | | | 4 | 2% |
| Rather decreased |  | | | 19 | 8% |
| Not changed |  | | | 165 | 69% |
| Rather increased |  | | | 44 | 18% |
| Increased |  | | | 6 | 3% |
| **Examples for decreased dosages (optional):** | 3 | | |  |  |
| *Vancomycin* |  | | | 1 |  |
| *Cefuroxim DANI* |  | | | 1 |  |
| *Beta-lactam antibiotics (Pip/Tazo & Meropenem)* |  | | | 1 |  |
| **Examples for increased dosages (optional):** | 14 | | |  |  |
| *Vancomycin* |  | | | 2 |  |
| *Cefuroxime* |  | | | 2 |  |
| *Cephalosporins* |  | | | 1 |  |
| *Piperacillin/Tazobactam* |  | | | 2 |  |
| *Penicillin* |  | | | 2 |  |
| *Ertapenem* |  | | | 1 |  |
| *Beta-lactams* |  | | | 1 |  |
| *Tigecycline* |  | | | 1 |  |
| *Imipenem* |  | | | 1 |  |
| *Carbapenems* |  | | | 1 |  |
| **23. Due to the ISGs recommendations the durations of antimicrobial therapy you are prescribing have in tendency…** | 238 | | |  |  |
| Decreased |  | | | 10 | 4% |
| Rather decreased |  | | | 72 | 30% |
| Not changed |  | | | 136 | 57% |
| Rather increased |  | | | 19 | 8% |
| Increased |  | | | 1 | 0,4% |
| **Examples for decreased duration (optional):** | 2 | | |  |  |
| *Imipenem* |  | | | 1 |  |
| *Piperacillin/Tazobactam* |  | | | 1 |  |
| **Examples for increased duration (optional):** | 1 | | |  |  |
| *Piperacillin/Tazobactam* |  | | | 1 |  |
| **24. Due to the ISGs recommendations you are switching patients more commonly / quicker from i.v. to oral antimicrobial therapy…** | 238 | | |  |  |
| Not true |  | | | 50 | 21% |
| Rather not true |  | | | 34 | 14% |
| Don't know |  | | | 117 | 49% |
| Rather true |  | | | 29 | 12% |
| True |  | | | 8 | 3% |
| **Examples rather i.v. (optional):** | 10 | | |  |  |
| *Cefotaxim* |  | | | 4 |  |
| *Cefuroxime* |  | | | 1 |  |
| *Cephalosporins* |  | | | 1 |  |
| *Penicillins* |  | | | 1 |  |
| *Ampicillin/Sulbactam* |  | | | 1 |  |
| **Examples rather oral (optional)** | 2 | | |  |  |
| *Fluoroquinolones* |  | | | 2 |  |
| **25. Do you find the ISGs user-friendly?** | 238 | | |  |  |
| Yes |  | | | 228 | 96% |
| No |  | | | 10 | 4% |
| **26. Which platform is most usable for you in your daily clinical routine?** | 238 | | |  |  |
| A pocket guide |  | | | 95 | 40% |
| An app |  | | | 108 | 45% |
| A PDF on your ward computer |  | | | 35 | 15% |
| **27. How well is the app functioning on your smartphone?** | 110 | | |  |  |
| Bad |  | | | 0 | 0% |
| Rather bad |  | | | 8 | 7% |
| Rather good |  | | | 45 | 41% |
| Good |  | | | 57 | 52% |
| **28. Would you say the price of 3,49 € for the app is justified?** | 110 | | |  |  |
| Yes |  | | | 89 | 81% |
| No |  | | | 21 | 19% |
| **29. Did you not download the app because it was not for free?** | 128 | | |  |  |
| Yes |  | | | 46 | 36% |
| No |  | | | 82 | 64% |
| **30. Do you have suggestions for improvements of the mobile app?** | 28 | | |  |  |
| Summary of written comments: |  | | |  |  |
| *Better search function* |  | | | 8 |  |
| *App for free* |  | | | 11 |  |
| *Color code system* |  | | | 2 |  |
| *Table of content at start* |  | | | 1 |  |
| *quick access buttons* |  | | | 1 |  |
| **31. Do you have any other comments regarding the guidelines or this study?** | 31 | | |  |  |
| *Summary of frequently made comments:* | | |  |  |  |
| *More recommendations regarding oral antibiotic therapy.* | | |  | *5* |  |
| *The app should be provided free of charge for employees* | | |  | *3* |  |
| *Table of content in alphabetical order.* |  | | | *2* |  |
| *Great work/ thank you for the ISGs.* |  | | | *8* |  |
| *I couldn’t observe changes in prescribing behavior as I use the ISGs since I started working.* |  | | | *2* |  |
| *Suggestion to add recommendations/ content on:*   - *Intracranial infections* - *Dosing under CRRT* - *Dosing of perioperative prophylaxis in obese patients* - *Oral medication of ENT outpatients* - *Extension of recommendations for the emergency room* - *Use of Surveillance blood cultures* - *More diagnostic criteria and indications for antibiotic treatment* - *Spectrum of efficacy of antibiotics* | | |  |  |  |
