# Supplementary material for: Assessing the impact of institution-specific guidelines for antimicrobials on doctors’ prescribing behavior at a German tertiary-care center and the additional benefits of providing a mobile application
Source: PLoS One. 2020 Nov 3;15(11):e0241642. doi: 10.1371/journal.pone.0241642 (PMC7608892; doi:10.1371/journal.pone.0241642)
Supplement: S2 Table — Answers to question 17, translated from German. (DOCX) [file pone.0241642.s002.docx]

**Supporting information**

**S2 Table. Written comments of survey participants regarding disagreement with ISG**

**recommendations (survey item 17)**

| **Written comments clustered by topics (translated from German):** | |
| --- | --- |
| *Adnexitis recommendation* | |
| *Adnexitis* | |
| *Spondylodiscitis* | |
| *Therapy duration for spondylodiscitis* | |
| *Intracerebral infections* | |
| *Therapy duration for intracranial infections* | |
| *Lack of specific recommendations for neurosurgical infections* | |
| *Ampicillin: dose adjustment in chronic kidney disease in therapy of Meningitis/Listerial meningitis* | |
| *Meningitis* | |
| *Antibiotic therapy of meningitis seems in selected cases to be dosed too high (Situations with limited cell count, low risk of bacterial genesis, not differentiated enough)* | |
| *Nosocomial Meningitis/ device associated CSF-effect: primary carbapenem-therapy instead of cephalosporins are in my opinion an overtherapy* | |
| *Meningitis: discontinuation of Dexamethasone when no proof of* | |
| *Meningococci; Ampicillin and Meropenem as perfusor instead of intermittendly* | |
| *Intrinsic resistance of Enterococci for gastro intestinal infections* | |
| *Guideline for bile tract infections: among our patients with biliary sepsis there is a relevant proportion of enterococcal infections, therefore the combination cefotaxim/metronidazol is problematic* | |
| *UTI: after multiple consultations with urology recommendation of Ciprofloxacin* | |
| *page 111: UTI: Cefixim is not available, Ampicillin/Sulbactam for E. coli usually only intermediate in the antibiogram* | |
| *oral alternatives for pyelonephritis* | |
| *transrectal prostate biopsy* | |
| *Possibly reevaluation of dose adjustment for chronic kidney disease --> risk of underdosing? For instance no renal dose adjustment for betalactam antibiotics in the first 48h or the like.* | |
| *For zoster ophthalmicus therapy we usually use at maximum 7.5mg/kg bodyweight, since that is enough in the vast majority of cases, escalation is still possible and the higher dose causes more side effects.* | |
| *For zoster ophthalmicus without eye involvment and in otherwise healthy patients a dose of 5-7.5mg/kg bodyweight is sufficient.* | |
| *We usually treat phlegmones with Ampicillin/Sulbactam + Clindamycin because of the increased tissue penetration as opposed to only Ampicillin and Clindamycin* | |
| *HAP: Aminopenicillin/Betalactam inhibitor or 3a Cepahlosporin; for increased risk of multidrug resitance Piperacellin/Tazobactam or Carbapenem* | |
| *too few oral alternatives* | |
| *sometimes not enough recommendations, e.g. when multible drug intolerances or other contraindications are present* | |
| *page 97 repetition in surgeries is contradictory ("1-2fold halflife" vs. e.g. after "3-4h" for Cefuroxim (halflife 70min))* | |
| *Carbapenems as perioperative prophylaxis in rectal surgery* | |
| *Choice of substances, application i.v./oral* | |
| *Clindamycin 3 x 600mg for necrotising fasciitis (dosage too low) and Vancomycin orally for Clostridium difficile infections (initial dose too high)* | |
| *Dose recommendations for neonatological infections are not granular enough, time gaps have to be dependent on the gestational age and the live age.* | |
