# Supplementary material for: Assessing the impact of institution-specific guidelines for antimicrobials on doctors’ prescribing behavior at a German tertiary-care center and the additional benefits of providing a mobile application
Source: PLoS One. 2020 Nov 3;15(11):e0241642. doi: 10.1371/journal.pone.0241642 (PMC7608892; doi:10.1371/journal.pone.0241642)
Supplement: S3 Table — Answers to question 31 (free text, translated from German). (DOCX) [file pone.0241642.s003.docx]

**Supporting information**

**S3 Table. Written comments of survey participants regarding the ISGs or the study**

**(survey item 31)**

| **Comments regarding ISG content:** |
| --- |
| *Inclusion of spectrum of efficacy of antibiotics would be nice.* |
| *More oral alternatives for common problems like urinary tract infections, respiratory infections, thrombophlebitis.* |
| *Inclusion of dosing under CRRT (continuous renal replacement therapy) on the intensive care unit.* |
| *Inclusion of a chapter regarding intracranial infections/ infections in neurosurgery e.g. brain abscess.* |
| *Linking of all dose recommendations for special cases. Direct link from substances to respective dose adjustments i.e. for reduced kidney function and for obese patients.* |
| *Recommendations for dosing in perioperative prophylaxis in obese patients. Weight adaption?* |
| *For ENT-infections there is a lack of information for oral medications for outpatients. First choice are almost exclusively intravenous medications which is not applicable for many cases that are managed as outpatients. More information regarding oral antibiotic therapy would be great.* |
| *For many ENT-infections only i.v. recommendations. For outpatients it would be great if there were oral alternatives for all indications or the remark that an intravenous application is absolutely essential.* |
| *Even more critical respectively more conservative indication of antibiotic prescribing.* |
| *Recommendation regarding surveillance blood cultures.* |
| *Shortening of antibiotic treatment for instance for HAP with clinical recovery.* |
| *Rotation of antibiotic regimens (e.g. different cephalosporins)* |
| *Recommendation for continued administration for instance ampicillin* |
| *Conflicting recommendations for the calculated antibiotic treatment for bacterial endocarditis: recommendations in the guideline differ from those in cardiological recommendations and in part from those given on the microbiological ward round.* |
| *Important recommendations for empirical antibiotic choices in the ER could be extended.* |
| *More alternative therapy schemes would be nice, especially for reoccurring pneumonia and sepsis.* |
| *More alternative antibiotics. More in-depth information for mycology and virology.* |
| *Oral and maxillofacial surgery and ENT treat orbital phlegmones typically with Unacid and not Tazobac as recommended in the guideline.* |
| *More diagnostic criteria and indications would be helpful, though this might go beyond the scope of the ISG.* |
| *When should antibiotic courses be extended or shortened?* |
| *After what duration of an intravenous therapy is a switch to an oral therapy possible or even recommended. What substances should be switched to? Neither Patients nor wards like to “wait” 3-4 days for the “completion” of the therapy. Many thanks for the guideline!* |
| *Therapy recommendations for postoperative wound infections after dermatologic surgeries. Cefuroxim has a poor bioavailability and should therefore applied intravenous which may increase the duration of stay.* |
| **Comments regarding ISG structure:** |
| *Alphabetical table of content would be desirable but the current set up works.* |
| *For the pocket guide: a table of content for the infections in alphabetical order would be helpful. Great work, carry on!* |
| *The book could be clearer organized, for instance with information on the sides. Otherwise great book.* |
| **Comments regarding the study:** |
| *As I am currently not working in direct patient care and therefore not prescribing antibiotics some questions don’t apply to me. I still had to answer them.* |
| *There might be a strong bias: Who cares little for surveys may also act differently in regards to the guidelines.* |
| *I use the ISG since I started working, therefore I cannot note any changes in my behavior.* |
| *When using the guideline since the beginning of the work in the hospital some questions are difficult to answer since it’s hard to judge my behavior without the ISG.* |
| **Other comments:** |
| *An outstanding project, exemplary implemented!* |
| *A fantastic offer! It’s great that microbiologists, infectious disease doctors, virologists and pharmacists can be called any time regarding complex cases and that weekly microbiological ward rounds are happening on ID – high risk wards. Many thanks!* |
| *Very useful manual. Carry the pocket guide always in my lab coat. Quick and clear reference.* |
| *Especially helpful for wards like psychiatry where antibiotic treatment is not part of the daily clinical routine.* |
| *Really very helpful.* |
| *The guideline is outstanding.* |
| *Use of the IGS in Pathology: To look up whether an antibiotic therapy of a deceased patient was indicated. Especially whether associated side effects can be seen.* |
| *Given the rare prescription of antibiotics in outpatient dental medicine outside of the pain clinic and the few indications the IGS are used infrequently but provide a good basis.* |
| *App should be provided for free by the employer (as a matter of principle).* |
| *Please provide free of charge for employees!* |
| *I think the price of the app is justified, yet I think that employees should get the app for free.* |
