# Supplementary material for: Assessing the impact of institution-specific guidelines for antimicrobials on doctors’ prescribing behavior at a German tertiary-care center and the additional benefits of providing a mobile application
Source: PLoS One. 2020 Nov 3;15(11):e0241642. doi: 10.1371/journal.pone.0241642 (PMC7608892; doi:10.1371/journal.pone.0241642)
Supplement: S1 Additional figures — (DOCX) [file pone.0241642.s004.docx]

**Supporting Information**

**S4. Additional Figures**

**Intravenous/oral sequential therapy**

**Reported rate of ISG use as a percentage of total prescriptions**

**Reported adherence to ISGs**
